# Safety of psychotropic medications in people with COVID-19

**DOI:** 10.1192/j.eurpsy.2021.274

**Published:** 2021-08-13

**Authors:** G. Ostuzzi, D. Papola, C. Gastaldon, C. Barbui

**Affiliations:** 1 Department Of Neuroscience, Biomedicine And Movement Sciences, University of Verona, Verona, Italy; 2 Neuroscience, Psychological And Psychiatric Science, Science Of Bio Movement, University of Verona, Verona, Italy

**Keywords:** coronavirus, COVID-19, Psychopharmacology, drug-drug interaction

## Abstract

**Introduction:**

People with coronavirus disease (COVID-19) may frequently require treatment with psychotropic medications, but the underlying medical condition and possible interaction with medical treatments might pose serious safety issues.

**Objectives:**

To review the direct and indirect evidence on the safety of psychotropic drugs in people with COVID-19 and provide practical recommendations for frontline clinicians.

**Methods:**

An international, multi-disciplinary working group was established with the aim of producing evidence-based recommendations on the management of psychotropic medications in people with COVID-19, following the WHO Rapid Advice Guidelines methodology in the context of a public health emergency. Evidence retrieved was focused on the risk of respiratory, cardiovascular, infective, hemostatic, and consciousness alterations related to the use of psychotropic medications. Furthermore, drug-drug interactions between psychotropic and medical treatments used in people with COVID-19 was reviewed and critically discussed by the working group.

**Results:**

The analysis of available evidence, although indirect, showed that all classes of psychotropic medications might carry relevant safety risks for people with COVID-19. The working group produced a set of 12 recommendations to support clinicians in the assessment of the anticipated risk of psychotropic-related unfavourable events, and how to practically manage this risk, including when it is appropriate to avoid, withdraw, switch, or adjust the dose of the medication.

**Conclusions:**

The present evidence-based recommendations will improve the quality of psychiatric care in people with COVID-19, allowing an appropriate management of the medical condition without worsening the psychiatric condition and vice versa.

**Disclosure:**

No significant relationships.

